# Apathy in Parkinson’s Disease: Diagnosis, Neuropsychological Correlates, Pathophysiology and Treatment

**DOI:** 10.3233/BEN-129025

**Published:** 2013

**Authors:** Gabriella Santangelo, Luigi Trojano, Paolo Barone, Domenico Errico, Dario Grossi, Carmine Vitale

**Affiliations:** ^1^Department of Psychology, Second University of Naples, CasertaItaly; ^2^Istituto di Diagnosi e Cura “Hermitage Capodimonte”, NaplesItaly; ^3^Neurodegenerative Diseases Center, University of Salerno, SalernoItaly; ^4^University of Naples “Parthenope”, NaplesItaly

**Keywords:** Parkinson’s disease, depression, apathy, non-motor symptoms, frontal/executive functions

## Abstract

Apathy has been defined as lack of motivation. It has been traditionally considered as a symptom of psychiatric disorders, such as major depression and schizophrenia, but more recently it has been recognized as a specific neuropsychiatric syndrome associated with neurodegenerative such as Parkinson’s disease (PD). As a consequence the reported prevalence of apathy in PD ranges from 13.9% to 70%; the mean prevalence is 35%. Prevalence of “pure apathy” (i.e., of apathy without comorbid depression and dementia) seems to be substantially lower, from 3 to 47.9%. High levels of apathy in PD are associated with decreased daily function, specific cognitive deficits and increased stress for families. Although neuroimaging studies do not provide a unique anatomic pattern, several data suggest that the ventromedial prefrontal cortex and the basal ganglia connected through frontal-subcortical circuits, are particularly involved in the genesis of apathy. At present, there are no approved medications for the treatment of apathy in and no proof of efficacy exists for any drug in current use. Further studies and innovative pharmacologic approaches are thus needed to ameliorate our understanding and treatment of apathy in PD.

## 1. Introduction

Apathy has been defined as "lack of motivation not attributable to diminished level of consciousness, cognitive impairment or emotional distress" by Marin [1, 2]. It clinically manifests as: 1) reduced goal directed behavior, 2) reduced goal directed cognition and 3) reduced emotional concomitants of goal directed behaviors. Marin provided an operational definition of each aspect of apathy: the reduced goal directed behavior was defined as lack of effort, initiative and productivity; the reduced goal directed cognition as decreased interests, lack of plans and goals, and lack of concern about one's own health or functional status; and finally, the reduced emotional concomitants of goal directed behaviors as flattened affect, emotional indifference and restricted responses to important life events [1].

More recently, Levy and Dubois [3] criticized Marin's definition of apathy as "lack of motivation" and proposed an alternative approach. They defined apathy as an observable and quantifiable behavioral syndrome characterized by a quantitative reduction of self-generated voluntary and purposeful behaviors despite the patient's contextual or physical changes. In other terms, apathy may be considered as a pathology of voluntary action or goal-directed behavior (GBD) and it may arise from alterations occurring at the level of elaboration, execution and/or control of GBD [4].

Levy and Dubois [3] proposed to divide apathetic syndromes into three subtypes on the basis of specific

ISSN 0953-4180/13/$27.50 © 2013 – IOS Press and the authors. All rights reserved type (i.e. emotional, cognitive or behavioral) of process disrupted during the completion of GDB: 1)'emotional', 2) 'cognitive' and 3) 'auto-activation' apathy. The first subtype of apathy is related to disruption of 'emotional' processing and refers to a reduction in GDB arising from an inability to link affective and emotional signals with ongoing and forthcoming behaviors. This subtype of apathy appears as either loss of will, loss of goals or emotional blunting or as diminution of one's ability to evaluate the consequences of future actions [5]. The cognitive apathy is related to disruption of 'cognitive' processing and may be called cognitive inertia: it refers to the reduction in GDB arising from alterations of cognitive functions (e.g. executive functions) needed to elaborate the plan of actions. Finally, the third subtype of apathy is related to an 'Auto-activation' deficit: it refers to difficulties in activating thoughts or initiating the motor program needed to complete the behavior. Patients with this subtype show severe apathy, characterized by difficulties in self-initiating actions or thoughts ('mental emptiness'), contrasting with relatively spared, externally driven responses.

The apathy can appear as a symptom of psychiatric disorders, such as major depression and schizophrenia, but more recently it has been recognized as a specific neuropsychiatric syndrome arising from dysfunctions of prefrontal cortex and basal ganglia connected through fronto-subcortical circuitries [3,6]. In fact, apathy is reported in the context of several neurological disorders in which altered functioning of prefrontal cortex and/or basal ganglia occurs, such as stroke [7], Alzheimer's disease [8], Huntington's disease [9–11], or progressive supranuclear palsy [12–14]. The neurologic disease in which apathy has been most intensely investigated is Parkinson's Disease (PD). This article offers a review of clinical and neuropsychological correlates, diagnostic strategies, etiologic mechanisms and treatment of apathy in patients with PD.

## 2. Prevalence of apathy in PD

In recent years strong efforts have been made to recognize apathy in PD and to define how common it is. After the pioneering study by Starkstein et al. [15], frequency of apathy has been investigated by many authors (see Table 1). The prevalence rate of apathy in PD ranges from 13.9% to 70%; the mean prevalence is 35% (see Table 1). One important factor contributing to the wide variability across studies is represented by different recruitment criteria: early studies assessed prevalence including apathetic patients with depression or dementia, whereas more recently prevalence of "pure apathy" (i.e., apathy without comorbid depression and dementia) has been found to range from 3 to 47.9% (Table 1). Other possible causes of divergent data about prevalence of apathy are type of population studied (patients recruited from movement disorder units vs patients recruited from the community), and diagnostic/screening tools (self-report vs caregiver version of tools or diagnostic criteria). In detail, most prevalence studies were hospital-based and included patients recruited from outpatient neurological clinics [15,17–22,24,27,29–31,33–39]; in these studies the prevalence rate of apathy ranges from 17.2 to 70. The few community-based prospective studies [16,23, 25,26,28,32] tend to report lower prevalence. Recently, Pedersen et al. [25] using the motivation/initiative item of UPDRS section I, found a prevalence of 37.9% (88/232 PD patients), whereas pure apathy was found in 9.1% of cases. Using more restrictive criteria (apathy subscale of Neuropsychiatric Inventory (NPI) and diagnostic consensus criteria for apathy [42] validated in PD [31]), Pedersen et al. [32] reported a prevalence of 22.9% (40 patients), in 175 non-demented, drug-naïve patients with newly diagnosed PD, with a prevalence of "pure apathy" of 14.3%. A 4 year-follow-up study [26], reported apathy in PD as a persistent behavioral feature, with an annual incidence rate of 12.3%: in other terms, occurrence of apathy increased substantially and affected more than 60% at the last follow-up. Taken together, these findings demonstrated that apathy may be considered as a non motor symptom of PD occurring since early stages of disease, and increasing in frequency as the disease progresses.

## 3. Assessment and phenomenology

According to diagnostic criteria proposed by Starkstein and Leentjens [43] apathy includes both loss of motivation and loss of emotionality. Subsequently, these diagnostic criteria have been revised by a task force commissioned by the European Alzheimer's Disease Consortium (EADC; see Table 2) [42]. In a recent validation study on a PD sample [31] EADC criteria proved to show good acceptability, internal consistency, concurrent validity, and moderate to good discriminant validity with depression. These criteria identified apathy in 17.2% of the PD sample, a rate in the lower part of abovementioned range. Therefore, EADC diag-

Table 1

Prevalence (%) of apathy and pure apathy in Parkinson's disease

| Authors | Sample of PD patients | Population | Depression | Dementia | Prevalence (%) | "Pure apathy" (i.e. without depression and dementia) (%) | Tools for apathy |
|---|---|---|---|---|---|---|---|
| Starkstein et al. [15] | 50 | Tertiary clinic PD | Not excluded | Not excluded | 32 | 12 | AS |
| Aarsland et al. [16] | 139 | Epidemiological study | Not excluded | Not excluded | 16.5 | | Apathy sub scale of NPI |
| Isella et al. [17] | 30 | Tertiary clinic PD | Not excluded | Excluded | 70 | | AS |
| Pluck and Brown [18] | 45 | Tertiary clinic PD | Not excluded | Not exclude | 36 | | AES |
| Kirsch-Darrow et al. [19] | 80 | Tertiary clinic PD | Not excluded | Not excluded | 51 (28.8% of apathy without depression) | | AES |
| Aarsland et al. [20] | 537 | Tertiary clinic PD | Excluded | Not excluded | 54 | | NPI |
| Dujardin et al. [21] | 159 | Tertiary clinic PD | Not excluded | Not excluded | 32.1 | 6.9 | LARS |
| Kulisevsky et al. [22] | 1351 | Tertiary clinic PD | Not excluded | Excluded | 48.3 | | Apathy sub scale of NPI |
| Pedersen et al. [23] | 89 | Epidemiological study | Not excluded | Not excluded | 17 | | Specific scheme diagnostic [40,41] |
| Kirsch-Darrow et al. [24] | 301 | Tertiary clinic PD | Not excluded | Not excluded | 50 (31.4% of apathy without depression | | AS |
| Pedersen et al. [25] | 232 | Epidemiological study | Not excluded | Not excluded | 37.9 | 9.1 | UPDRS section I |
| Pedersen et al. [26] | 79 | Epidemiological study | Not excluded | Not excluded | 13.9% at baseline; 63.3% at follow-up | | Apathy sub scale of NPI |
| Starkstein et al. [27] | 164 | Tertiary clinic PD | Not excluded | Not excluded | 32 | 3 | diagnostic criteria for based on Marin et al. [1] |
| Aarsland et al., 2009 [28] | 175 | Epidemiological study | Not excluded | Excluded | 27.4 | | Apathy sub scale of NPI |
| Reijnders et al. [29] | 55 | Tertiary clinic PD | Excluded | Excluded | | 16.4 (by LARS) 12.7 (by AES) 32.7 (by NPI) | LARS, AES, apathy subscale of NPI |
| Leiknes et al. [30] | 189 | Tertiary clinic PD | Not excluded | Not excluded | 29.1 | | Apathy sub scale of NPI |
| Drijgers et al. [31] | 122 | Tertiary clinic PD | Not excluded | Excluded | 17.2 | 8.2 | Diagnostic criteria [42]; Lille Apathy Rating Scale (LARS) and the apathy section of the Neuropsychiatric Inventory (NPI). |
| Pedersen et al., 2010 [32] | 175 | Epidemiological study | Not excluded | Excluded | 22.9 | 14.3 | NPI, diagnostic criteria for apathy [42] |
| Kirsch-Darrow et al. [33] | 161 | Tertiary clinic PD | Not excluded | Excluded | 33.5 | 17.4 | AS |
| Varanese et al. [34] | 48 | Tertiary clinic PD | Excluded | Excluded | 47.9 | 47.9 | AES |
| Leroi et al. [35] | 71 | Tertiary clinic PD | Not excluded | Excluded | 31 | | AES |
| Leroi et al. [36] | 99 | Tertiary clinic PD | Not excluded | Excluded | 26.3 | | AS |
| Ziropadja et al. [37] | 360 | Tertiary clinic PD | Not excluded | Not excluded | 60.2 | 17.5 | AS |
| Benito Leòn et al. [38] | 557 | Tertiary clinic PD | Not excluded | Excluded | 52.2 | 21.7 | LARS |
| Cubo et al. [39] | 557 | Tertiary clinic PD | Not excluded | Excluded | | 33.4 | LARS |

AS, Apathy Scale; AES, Apathy Evaluation Scale, LARS, Lille Apathy Rating Scale; NPI, Neuropsychiatric Inventory, PD, Parkinson's Disease.

Table 2
Diagnostic criteria for apathy (adapted from [42])

*For a diagnosis of apathy the patient should fulfill the criteria A, B, C and D:*

*Criterion A.* Loss of or diminished motivation in comparison to the patient's previous level of functioning and which is not consistent with his age or culture. These changes in motivation may be reported by the patient himself or by the observations of others.

*Criterion B.* Presence of at least one symptom in at least 2 of the 3 following domains for a period of at least 4 weeks and present most of the time.

*B1. Domain behaviour.* Loss of, or diminished, goal-directed behaviour as evidenced by at least one of the following:
- Initiation symptom: loss of self-initiated behaviour (e.g. starting conversation, doing basic tasks of day-to-day living, seeking social activities, communicating choices);
- Responsiveness symptom: loss of environment-stimulated behaviour (e.g. responding to conversation, participating in social activities)

*B2. Domain cognition.* Loss of, or diminished, goal-directed cognitive activity as evidenced by at least one of the following:
- Initiation symptom: loss of spontaneous ideas and curiosity for routine and new events (i.e, challenging tasks, recent news, social opportunities, personal/family and social affairs);
- Responsiveness symptom: loss of environment-stimulated ideas and curiosity for routine and new events (i.e, in the person's residence, neighbourhood or community).

*B3. Domain emotion.* Loss of, or diminished, emotion as evidenced by at least one of the following:
- Initiation symptom: loss of spontaneous emotion, observed or self-reported (e.g. subjective feeling of weak or absent emotions, or observation by others of a blunted affect);
- Responsiveness symptom: loss of emotional responsiveness to positive or negative stimuli or events (for example, observer-reports of unchanging affect, or of little emotional reaction to exciting events, personal loss, serious illness, emotional-laden news)

*Criterion C.* These symptoms (A and B) cause clinically significant impairment in personal, social, occupational, or other important areas of functioning.

*Criterion D.* The symptoms (A and B) are not exclusively explained or due to physical disabilities (e.g. blindness and loss of hearing), to motor disabilities, to diminished level of consciousness or to the direct physiological effects of a substance (e.g. drug of abuse, a medication.

nostic criteria, that appear to be well suited for clinical practice and research, seem to be relatively conservative if compared to cut-off scores on currently available apathy rating scales [31].

In common clinical practice and research several questionnaires are now available for diagnosis of apathy in PD patients, and some of them encompass different versions to be compiled by clinician, by informant, or by patient as a self-rating scale. Presence and severity of apathy are often assessed by means of the apathy subscale included in NPI [16,20,22,26,28–32]. In most studies, however, apathy has been identified by means of self-report questionnaires, such as the Apathy Evaluation Scale (AES) [44], and its 14-item abbreviated form, the Apathy Scale (AS) [15,17–19,24,29,33–37], easier to be administered to PD patients. Using some items of the Apathy Scale and the standardized criteria adapted from Marin [1], an algorithm to diagnose apathy has been developed for Alzheimer's disease [45], and validated for PD [27].

Recently, a task force commissioned by the Movement Disorders Society (MDS) to compare several apathy and anhedonia scales in PD, concluded that item 4 of the Unified Parkinson Disease Rating Scale is useful only for "crude screening purposes", whereas the AS is "recommended" for the assessment of apathy in PD [46]. However, since the AS yields only a global score and thus provides little information about profiles of apathy, recently, a new tool has been developed, the Lille Apathy Rating Scale (LARS) [47], whose French and English versions have been specifically validated in PD patients [47,48]. The LARS is a semi-structured interview assessing four distinct dimensions of apathy: intellectual curiosity (IC), emotion (E), action initiation (AI), and self-awareness (SA). The LARS seems to provide information about different clinical manifestations (i.e., cognitive, behavioral, affective) of apathy [47] and represents a potentially useful instrument to characterize apathy in PD. Dujardin et al. [21], using the LARS, found that among its four dimensions (IC, E, AI and SA) AI and IC dimensions contributed strongly to severity of apathy in PD. Subsequently, using EADC diagnostic criteria [42], Drijngers et al. [31] found that reduced goal-directed behavior followed by reduced goal-directed cognition are the most frequently reported domains in apathetic PD patients; reduced spontaneous emotion seems to occur most often in combination with one or both of the other two domains, and its presence may signal more severe levels of apathy.

Since demented or severely apathetic PD patients may be unaware of their condition, use of self-report versions of available scales may result in underestimation of the symptom; for this reason, it is very important to debrief patients' caregivers, who usually are a reliable source of information. A specific informant-based version is included in the LARS (the LARS-i) [49]. Ideally, assessment of apathy in PD should include information collected from both the patient and the carer, and also take into account systematic observation from physician's perspective, possibly guided by a formalized inventory such as that suggested in a recent study [50].

## 4. Relationships between apathy and depression

Particular mention should be made of the relationships between apathy and depression, since apathy has been traditionally considered as an aspect of depression. The overlap between apathy and depression can cause substantial difficulties in identifying and distinguishing apathetic patients from depressed patients in PD [51,52]. Although most studies found an association between apathy and depression in PD patients [25,26, 32,34,37,38], and between apathy and anhedonia [18, 52,53], others showed that apathy may occur independently of depression in PD [17–19,33,39,52]. Apathetic PD patients can be misdiagnosed as depressed, and consequently treated with inadequate drugs [52]; therefore, although difficult, recognizing apathy and differentiating it from depression is important also in view of possible differentiated treatment strategies (see below).

Recently, Kirsch-Darrow et al. [33] confirmed that apathy and depression can dissociate in PD, and are characterized by different features: depression includes sadness and negative thoughts about the self, while diagnosis of apathy rests on lack of initiation and lack of effort, but not on negative self or event appraisal. In this perspective, Kirsch-Darrow et al. [33] analysed items from the Beck Depression Inventory (BDI-II) and the AS, and found that they can be combined to obtain four separate subscales ("pure" apathy, "pure" depression, overlapping symptoms of loss of interest and pleasure, and somatic symptoms) to differentiate specific symptoms related to apathy or depression. Moreover, in a recent longitudinal study, Zahodne et al. [54] found that apathy and depression show a different progression over time: apathy worsened following a linear evolution, whereas depressive symptoms tended to show a fluctuating course, with improvement and worsening over time. These findings further supported the dissociation between apathy and depression in PD.

## 5. Clinical and cognitive correlates of apathy in PD

Few studies investigated demographic and specific clinical correlates of apathy in PD. As for association between gender and apathy, some studies reported that apathy is more frequent in male than in female PD patients [17,21,32] but such findings were not confirmed by other studies [37,55]. The association between apathy and male gender might indirectly support the possible role of testosterone levels in pathophysiology of apathy in male parkinsonians [56] but could also suggest that female caregivers pay attention and tend to report negative affective symptoms more frequently than male caregivers [32].

Table 3
Studies reporting an association of apathy with demographic and clinical aspects of Parkinson's disease

| Clinical factor | Authors |
|---|---|
| Age | Benito-Leon et al. [38]<br>Ziropadja et al. [37]<br>Pedersen et al. [25] |
| Education | Cubo et al. [39]<br>Benito-Leon et al. [38]<br>Pedersen et al. [25] |
| Disease duration | Ziropadja et al. [37] |
| Severity of motor symptoms | Cubo et al. [39]<br>Benito-Leon et al. [38]<br>Pedersen et al. [32]<br>Dujardin et al. [55]<br>Pedersen et al. [25] |
| Axial impairment | Ziropadja et al. [37] |
| Right predominant PD motor laterality | Cubo et al. [39] |
| L-dopa dosage | Ziropadja et al. [37]<br>Pedersen et al. [25] |

L-dopa, Levodopa; PD, Parkinson's Disease.

Apathetic PD patients are often less educated and older, use higher daily levodopa doses, have longer disease duration and more severe parkinsonism than non-apathetic patients [21,25,32,37–39] (see Table 3), although some divergent findings have been reported [17, 32,33]. While the association of poor education and apathy remains to be investigated [39], the correlation between apathy and clinical aspects of PD does not necessarily entails that apathy and motor symptoms share the same neural bases, but might suggest that degenerative processes underlying the two kinds of symptoms develop in parallel as the disease progresses. Actually, several studies have reported an association between apathy and motor symptoms, but many others have underlined that apathy can manifest in early stages of the disease, before development of relevant motor disability [32]. Moreover, as it will be discussed below, some lines of evidence suggest that apathy seems to be related to alteration of non-dopaminergic circuits.

Recently, Cubo et al. [39] found that patients living in a rural environment, with lower comorbidity and motor impairment, higher education background, and left predominant PD motor laterality were at lower risk of suffering from apathy.

In a 4-year prospective population-based longitudinal cohort study, Pedersen et al. [26] reported that dementia at baseline and a more rapid decline in speech and axial impairment during follow-up were independent risk factors for incident apathy. Moreover, Dujardin et al. [55] found that dementia was more frequent and severe in apathetic PD patients than in non-apathetic PD patients, and that apathy may be a predictive factor for cognitive decline over time in non-demented, non-depressed PD patients. According to the authors [55], the association between apathy and cognitive decline might be ascribed to an alteration of nondopaminergic circuits, mainly of cholinergic circuits originating in the basal forebrain nuclei – the main source of cholinergic projections to the cortex- both in PD [57] and in AD and related dementias [58,59].

The relationship between specific cognitive deficits and apathy has received increasing attention. Studies using brief cognitive screening tests (Mini Mental State Examination or Mattis Dementia Rating Scale) [15–18,21,25,32,37] found an association between apathy and cognitive impairment or dementia [16,18,25,32]. In other studies, specific tests have been used to assess the following neuropsychological domains: frontal lobe functions, memory and visuospatial skills (Table 4) [15, 17,18,32,34,60].

A consistent finding was that apathetic patients performed worse than non apathetic patients on specific frontal tests tapping spatial planning and cognitive flexibility [15,17,18,32,34,60]. However, most of these studies investigated neuropsychological correlates of apathy in PD without excluding PD patients with dementia and/or depression. Only two studies considered dementia and depression as exclusion criteria and found that patients with "pure apathy" performed worse than non apathetic PD patients on frontal tasks, thus suggesting a strong association between "pure apathy" and frontal dysfunctions [34,55]. However, this issue should be further investigated.

In the domain of memory, some studies did not find significant differences between apathetic and non-apathetic PD patients on short-term and long-term memory tasks [17,32,60]. On the contrary, three studies revealed that apathetic patients performed worse than non-apathetic patients on the memory subtest of the Cambridge Cognitive Examination (CAMCOG) [18], on Grober and Buschke 16-item recall test [55], and on paired associative learning test [15]. However, in these studies patients with dementia or depression were not excluded. Only one recent study [34] evidenced that patients with "pure apathy" had more difficulties on recall and recognition tasks of the California Verbal Learning Test II; the authors interpreted this impairment as due to poor strategy implementation at the encoding and the recall stages rather than a primary memory disorder [34].

Few studies explored visuospatial functions in PD patients with apathy and reported divergent findings [18,32,60]. Pedersen et al. [32] using a silhouette recognition task and Pluck and Brown [18] using the praxis subtest of CAMCOG found an association between impaired visuoperceptive and visuoconstructive functions and apathy in PD, whereas one study did not find this relationship [60].

The inconsistency in findings about association between apathy and cognitive deficits (memory and frontal dysfunctions) may be accounted for by methodological differences: these studies used a number of different measures to assess apathy or cognitive functions and different inclusion and exclusion criteria. However, most studies converge in highlighting that apathy, and particularly "pure apathy", is particularly related to dysexecutive dysfunctions, and these observations are consistent with the possible neural underpinnings of apathy (see below).

## 6. Impact of apathy on patients' and caregivers' quality of life in PD

It is well-established that PD patients experience progressive loss of ability to perform activities of daily living due to their motor disturbances, but in recent years some studies have demonstrated that apathy *per se* is associated with a decrease in functional autonomy [17, 32,61].

Increasing evidence is also accumulating about the impact of apathy on quality of life (QoL) in PD patients. It is now becoming clear that apathy contributes to reduce QoL in both early and advanced stages of the disease [38,61–63]. For example, in the PRIAMO study [62], a multicenter Italian survey involving 1072 PD patients, apathy was the symptom most often associated with poor QoL; however, a limitation of that study was that apathy was only assessed using few questions with dichotomous (yes/no) answers, without using a validated instrument. One recent study, using the LARS for diagnosis of apathy, found that apathy is one of the major clinical determinants of reduced QoL in recently diagnosed PD patients [38]. These results suggest that apathy may be a marker of poor QoL in

Table 4

Studies exploring cognitive correlates of apathy in Parkinson's disease

| Authors | Sample | Dementia | Depression | Cognitive domain | Cognitive test | Results |
|---|---|---|---|---|---|---|
| Starkstein et al., 1992 [15] | 50 PD patients:<br>6 with apathy,<br>15 with apathy and depression,<br>13 with depression,<br>16 no depression, no apathy | Not excluded | Not excluded | Frontal functions, Attention, Memory | WCST, Phonological fluency, Trail Making Test B, Symbol-digit, Digit forward, Digit backward, Paired associate learning | Apathetic < non apathetic on Phonological fluency, Trail Making Test, Paired associate learning |
| Isella et al., 2002 [17] | 30 PD patients:<br>10 with low apathy (FT-PD),<br>10 with moderate apathy (ST-PD)<br>10 with high apathy (TT-PD).<br>25 healthy controls (NC) | Excluded | Not excluded | Memory, Frontal functions | Story recall, Spatial span, Rey Figure copy and delayed recall, Phonological fluency, Category fluency, EXIT | All PD groups < NC on memory tests; no significant difference among the three PD groups. On frontal tests TT-PD < NC, with several differences between PD groups on single tests |
| Zgaljardic et al., 2007 [60] | 32 patients:<br>14 PD with apathy<br>18 PD without apathy | Excluded | Not excluded | Working Memory, Frontal functions, Visuospatial functions | Backwards Spatial span and Digit span (from Wechsler Memory Scale-III), Phonological fluency and Category fluency (from D-KEFS), Stroop Test, Odd Man Out Test, Twenty Questions subtest, Brief Test of Attention, Visual Form Discrimination Test, MDRS | Apathetic < non apathetic on some frontal tests: Letter fluency, Category fluency, Conceptualization (from MDSR) |
| Pedersen et al., 2010 [32] | 175 drug-naive PD patients:<br>40 with apathy<br>135 without apathy | Not excluded | Not excluded | Memory, Visuospatial functions, Frontal functions | California verbal learning test, VOSP Cube, VOSP silhouettes, Category fluency, Stroop test | Apathetic < non apathetic on VOSP silhouettes, Category fluency, Stroop test |
| Dujardin et al., 2009 [55] | 40 PD patients:<br>20 with apathy<br>20 without apathy | Excluded | Excluded | Memory, Frontal functions | Forward and backward digit span, Grober & Buschke 16-item recall test, Word generation test: letter P, Symbol digit, Stroop word/color test, Word generation (animal categories; alternating letters T and V) | Apathetic < non apathetic on Immediate recall and Total free recall of Grober and Buschke 16-item recall test, and on Word generation tests |
| Pluck and Brown, 2002 [18] | 45 PD patients:<br>17 with high apathy<br>28 with low apathy | Not excluded | Not excluded | Memory, Language, Visuospatial functions, Frontal functions | CAMCOG, Phonological fluency, Category fluency, WCST, Stroop test | Apathetic < non apathetic on total score, language, memory, praxis, and calculation scores included in CAMCOG, Phonological fluency, Category fluency, WCST, Stroop test |
| Vanarese et al., 2011 [34] | 47 PD patients:<br>23 with apathy<br>24 without apathy | Excluded | Excluded | Memory, Working memory, Attention, Frontal functions | Digit and spatial span forward and backward, California Verbal Learning Test II, Digit symbol and Visual scanning test (from D-KEFS), WCST, Number and letter sequencing test (from D-KEFS) | Apathetic < non apathetic on California Verbal Learning Test II (short delay free recall, long delay free and long delayed cued, delayed recognition), spatial span backward, and WCST (total correct, non-perseverative errors, categories completed) |

PD, Parkinson's Disease; WCST, Wisconsin Card Sorting Test; EXIT, Executive Interview; D-KEFS, Delis Kaplan Executive Function System; MDRS, Mattis Dementia Rating Scale; CAMCOG, cognitive battery of the Cambridge Examination for Mental Disorders of the Elderly.

Table 5
Results of neuroimaging studies on apathy in Parkinson's disease

| Authors | Methods | # of Subjects | Apathy | Results |
|---|---|---|---|---|
| Isella et al. [17] | MRI | 26 PD patients | Self-rated AS | No correlation between any specific measure of frontotemporal atrophy and severity of apathy |
| Remy et al. [66] | PET | 8 PD patients with and 12 PD patients without episodes of major depression based on DSM-IV criteria. | AES | Apathy was inversely correlated with $^{11}$C-RTI-32 binding (dopamine and noradrenaline) in the ventral striatum bilaterally |
| Le Jeune et al. [67] | PET | 12 PD patients after deep brain stimulation (DBS) of the subthalamic nucleus | Clinical version of AES | Increase of metabolism was correlated with modified apathy scores in the right frontal lobe, middle Gyrus (BA 10) and inferior gyrus (BA 45 and 46). temporal lobe (fusiform gyrus, BA 20), and postcentral gyrus (BA 43) (Fig. 2). Decrease of metabolism was correlated with modified apathy scores in the bilateral cingulated gyrus (BA 31) and left middle frontal gyrus (BA 9). |
| Reijnders et al. [29] | 3 T MRI | 55 PD patients | Informant-AES-I, LARS, apathy subtest of NPI | Apathy scores were found to be correlated with low GM density in the right posterior cingulate (PC) gyrus, and the bilateral inferior frontal gyrus<br>High apathy scores were correlated with low gray matter density values in a number of cortical brain areas: the bilateral precentral gyrus (BA 4, 6), the bilateral inferior parietal gyrus (BA 40), the bilateral inferior frontal gyrus (BA 44, 47), the bilateral insula (BA 13), the right (posterior) cingulate gyrus (BA 24, 30, 31), and the right precuneus (BA 31). |
| Skidmore et al. [65] | resting fMRI | 15 PD patients | Caregiver and self-report LARS | Increasing severity of apathy is associated with increased normalized ALFF signal in the right middle orbital gyrus and in the subgenual cingulated bilaterally and with decreased activity in the left supplementay motor region, left inferior parietal lobule and in the left fusiform gyrus.<br>Decreased ALFF activity in the Supplementary Motor Cortex and increased activity in the Right Orbitofrontal Cortex, and the Right Middle Frontal Gyrus predicts apathy score. |
| Robert et al. [68] | PET | 45 PD patients without dementia or depression | AES | Positive correlation between apathy cerebral metabolism in the right inferior frontal gyrus (Brodmann area [BA] [47]), right middle frontal gyrus (BA 10), right cuneus (BA 18), and right anterior insula (BA 13). Negative correlation was found between apathy and cerebellar metabolism. |

fMRI, functional Magnetic Resonance; PET, Positron Emission Tomography; PD, Parkinson's Disease; AS, Apathy Scale; AES, Apathy Evaluation Scale; LARS, Lille Apathy Rating Scale; NPI, Neuropsychiatric Inventory.

recently diagnosed PD and might affect all spheres of patients' subjective health status.

Very recent studies also demonstrated that apathy is associated with increased caregiver burden in PD patients with and without dementia [30,35]. Caregiver burden refers to the physical, mental and socio-economic problems that arise from caring for an individual affected by a chronic and disabling disease, such as PD [64]. Leiknes et al. [30] found that apathy is the non-motor symptom most commonly associated with caregiver distress in PD.

## 7. Neuro-anatomical and functional correlates of apathy in PD

The neurobiology of apathy in PD is complex and probably involves several different pathophysiological mechanisms, depending on the stage of disease and comorbid neuropsychiatric conditions. Apathy has been associated with deficits of the prefrontal-basal ganglia circuits [3]. Despite the fact that apathy is among the major neuropsychiatric features of PD, only limited MRI and functional studies explored neural correlates of apathy in PD (Table 5).

Isella et al. [17] examined a consecutive series of patients affected by idiopathic PD using an extensive neuropsychological battery and Marin's Apathy Scale; PD patients underwent MRI scan, followed by linear measurement of several frontotemporal structures. Approximately 45% of the PD sample showed apathy; no specific pattern of frontal and/or temporal atrophy was found to be associated with apathy. This discrepancy between the neuropsychological and the neuroradiological findings might be due to the lack of sensitivity and/or accuracy of the morphometric linear technique. In fact, a MRI voxel-based morphometry (VBM) study [29], found that in PD patients high apathy scores were correlated with low gray matter density values in several cortical brain areas, such as bilateral precentral gyrus, bilateral inferior parietal gyrus, bilateral inferior frontal gyrus, bilateral insula, right posterior cingulate gyrus, and right precuneus. Based on the involvement of the cingulate and premotor cortices the authors suggested that "autoactivation" deficits could be associated with apathy in PD, in line with the results of earlier studies addressing apathy in patients with Alzheimer's disease or depressive disorder [29].

Skidmore et al. [65] examined the relationship between apathy, depression, severity of motor symptoms and amplitude of low frequency fluctuation (ALFF) in a resting state fMRI paradigm. Apathy score on caregiver version of the LARS was best predicted by ALFF signal in the left supplementary motor cortex, the right orbitofrontal cortex, and the right middle frontal cortex. On this basis, the authors suggested that apathy may be either an "active-avoidant" syndrome due to hyperactivity of the right orbitofrontal lobe, or a purely "amotivational" syndrome due to reduced activity of the supplementary motor cortex; these two factors may combine to varying degrees and explain the wide variability of conditions that cause apathy [65].

A PET study by Remy et al. showed that in PD patients with and without major depression, high apathy scores were associated with decreased C-RTI-32 binding (dopamine and noradrenaline) in ventral striatum bilaterally [66]. In another PET study [67] apathy scores after deep brain stimulation of the subthalamic nucleus were found to be significantly correlated with decreased glucose metabolism in the left middle frontal gyrus and in the bilateral posterior cingulated gyrus a region with a critical role in the regulation of negative emotions through the encoding of emotional significance of the stimuli. Moreover, increased apathy scores significantly correlated with increased metabolic activity in the right frontal lobe, postcentral gyrus, and temporal lobe. Very recently, the same authors also found that metabolism within bilateral posterior lobe of the cerebellum inversely correlated with the AES score, in a cohort of 45 non-demented and non-depressed PD patients. These results support the view of a topographic segmentation of the cerebellum, with some structures implicated in motivation and behavioral regulation [68].

It is worth mentioning that two of the above neurofunctional studies [65,68] supported the view that the neural bases of apathy can be distinguished from neural correlates of depression in PD patients, thus reinforcing the idea that the two disorders are dissociable.

In conclusion, although apathy has been explained in terms of dysfunction of segregated frontal-subcortical loops [3], results of structural and functional studies failed to provide a unique anatomical pattern underlying apathy in PD and should be reinterpreted in a model which privileges lack of segregation between these circuits especially those linking the ventromedial prefrontal cortex to related regions in the basal ganglia.

## 8. Treatment of apathy in PD

At present, there are no approved drugs for treatment of apathy and no proof of efficacy exists for any drug in current use. Pharmacologic agents most frequently administered to apathetic patients include dopaminergic drugs, acetylcholinesterase inhibitors, atypical antipsychotics and psychostimulants. Few studies have assessed efficacy of psychotropic treatment for apathy in PD, and no randomized controlled trials have been conducted [69].

Treatment of coexisting apathy with dopaminergic drugs or the glutamatergic antagonist amantadine has been reported with some clinical benefits, but no randomized and well-controlled study confirmed these observations [69–72]. Whether L-dopa treatment may improve apathy remains unclear. Czernecki et al. [73] investigated motivation and sensitivity to reinforcement in 23 non-demented and non-depressed PD patients and found that L-dopa treatment may improve the subjective evaluation of motivation in apathetic PD patients. The same authors [71] reported long-term (6 months) efficacy of the dopamine agonist ropinirole (1–18 mg/day) in an open-label study that included 8 PD patients who developed apathy after withdrawal from dopaminergic treatment following deep brain stimulation (DBS) of the subthalamic nucleus (STN-DBS).

A recent meta-analysis on randomized, double-blind, placebo-controlled trials of pramipexole [74] found 7 trials ($N = 1296$) in which part I of the UPDRS was employed as a secondary outcome measure: results of this meta-analysis suggested that pramipexole had a beneficial effect on mood and motivational symptoms in PD patients who did not have major depressive disorder [74].

Further evidence suggesting that dopaminergic pathways contribute to pathogenesis of apathy derived from studies on DBS in PD patients. STN-DBS can induce or exacerbate apathy in some parkinsonian patients [69, 75–77]: in some patients apathy increased in severity and frequency, probably due to discontinuation of dopaminergic treatment after surgery, whereas in other patients no changes or even improvement were observed after surgery [77]. Stimulation of the associative and limbic region of the subthalamic nucleus and related structures has been proposed to be related to onset or worsening of apathy after surgery [77,78].

The reported association between apathy and cognitive symptoms but not motor deficits [21] might suggest that non-dopaminergic circuits are related to mechanism of apathy in PD. Drugs known to affect one or more non-dopaminergic transmitter systems have also be evaluated, alone or in combination, within or across comorbidities. A randomized controlled trial exploring efficacy of atomoxetine, a selective norepinephrine reuptake inhibitor, for treatment of clinically significant depressive symptoms in PD patients, with apathy as a secondary outcome measure, showed no benefit for either depression or apathy [79]. An open, non-comparative clinical study to assess efficacy and safety of tricyclic tianeptine, including 18 depressed PD patients (assessed on the Hamilton and Beck scales), showed no efficacy on motivation deficits and apathy [80]. A significant improvement of apathy was reported in a PD patient after treatment with 10 mg of methylphenidate, a stimulant drug chemically related to amphetamine that inhibits dopamine uptake and activates the brainstem arousal system and cerebral cortex [81].

Finally, two ongoing clinical trials are evaluating efficacy of acetylcholinesterase and MAO-B inhibitors on apathetic symptoms in PD patients. The first is a randomized, double-blind, placebo-controlled, parallel-group, multicenter trial to evaluate efficacy and acceptability of a 6-month treatment with rivastigmine on apathy in 60 PD non-demented patients [ClinicalTrials.gov identifier: NCT00767091; http://clinicaltrials.gov/ct2/show/NCT00767091]. This study has been completed but results have not yet been provided and published. The second ongoing randomised placebo-controlled trial evaluates efficacy of rasagiline in patients with Parkinson's disease and symptoms of apathy [ClinicalTrials.gov identifier: NCT00755027; http://clinicaltrials.gov/ct2/show/NCT00755027]. The primary outcome measure will be the mean change from baseline to study endpoint (week 12) in apathy scores as measured by the LARS and the Apathy Scale.

## 9. Conclusions

The large number of studies reviewed here witnesses that apathy cannot be considered anymore as a component of other psychiatric disturbances but is *per se* a cardinal non-motor features of PD, with a strong impact on patients' and their carers' wellbeing. Apathy can manifest since early stages of the disease and tends to increase its prevalence and severity in later stages, although this does not imply a direct link with progression of motor disturbances. The studies reviewed here provided divergent findings on many aspects, but the increasing awareness that apathy can manifest as a "pure" disorder has led researchers to select for their studies patients without depression or general cognitive impairment.

The most recent studies on "pure" apathy offered some convergent data about the relationships of apathy with cognitive dysexecutive deficits, in line with the hypothesis that specific neural underpinnings of apathy have to be searched for in a dysfunction of prefrontal cortex, and of the related cortico-subcortical circuits. However, available brain structural and metabolic studies failed to provide a clear pattern of neural correlates of apathy in PD. Likely, a broader approach, including for instance assessment of patients' personality traits, living environment, quality of supports and other relevant possible contributing motor and psychiatric factors, will improve understanding of apathy in PD.

Despite apathy is increasingly recognized as a source of significant disability in PD patients, no adequate trials have been conducted and no medication has been approved for this disorder. Additional studies are thus needed to address the contribute of the dopaminergic and non-dopaminergic systems in the pathogenesis of apathy in PD and to ameliorate treatment strategies.
